# A peculiar case of intususception in a pregnant woman: A diagnostic challenge

**DOI:** 10.1016/j.radcr.2023.05.048

**Published:** 2023-06-09

**Authors:** Firdaus Hayati, Asyraf Mohd Zuki, Ming Chin Lim, Pradeep Chand Chandran, Nornazirah Azizan, Mohamed Arif Hameed Sultan, Muhamad Hud Muhamad Zin, Khasnizal Abdul Karim

**Affiliations:** a Department of Surgery, Faculty of Medicine and Health Sciences, Universiti Malaysia Sabah, Jalan UMS, Kota Kinabalu, Sabah, 88450, Malaysia; b Department of Surgery, Faculty of Medicine, University of Malaya, Kuala Lumpur, Malaysia; c Department of Surgery, Queen Elizabeth Hospital, Ministry of Health Malaysia, Kota Kinabalu, Sabah, Malaysia; d Department of Pathology and Microbiology, Faculty of Medicine and Health Sciences, Universiti Malaysia Sabah, Kota Kinabalu, Sabah, Malaysia; e Department of Surgery, Teluk Intan Hospital, Ministry of Health Malaysia, Teluk Intan, Perak, Malaysia

**Keywords:** Abdominal neoplasms, case report, Cecal diseases, Ileal diseases, Intussusception, Pregnant

## Abstract

Adult intussusception presents a diagnostic challenge given its non-specific symptoms. It is not as common as in infants and young children. Traditionally, diagnostic steps are invariably fit for normal adults, but not in pregnancy which faces certain limitations. A 40-year-old pregnant mother, gravida 9 para 8 at 34-week gestation, complained of intermittent epigastric pain for 2 days, requiring hospitalization. She soon developed minimal per rectal bleeding that was ruled out as hemorrhoid. Imaging was limited due to her pregnancy status. She later developed spontaneous delivery to a premature baby. Computed tomography (CT) revealed an ileocolic intussusception, which was confirmed via exploratory laparotomy. Histology was consistent with inflammatory fibroid polyp. Acute abdomen in pregnancy can be due to various causes, thus a high index of suspicion and early CT abdomen might help in early diagnosis and treatment. The benefit of doing CT on the mother and the risk of CT on the fetus is to be weighed as the timely diagnosis can prevent bowel ischemia and reduce maternal morbidity and mortality. Surgery remains the definite management in adult intussusception and an exact diagnosis can be made during the operation.

## Introduction

Intussusception is one of the most prevalent pediatric surgical emergencies in infants and young children [1]. It is however considerably rare in adults. It occurs when a segment of the bowel invaginates into a more distal segment, which may cause intestinal obstruction and ultimately bowel ischemia [2,3]. Intussusception requires immediate treatment following adequate initial resuscitation, typically by an enema in children, then with surgery if unsuccessful enema reduction. In adults, surgical removal of the affected bowel is more frequently required.

Adult intussusception presents a diagnostic challenge given its nonspecific symptoms. However, the subsequent steps include clinical examination, biochemical investigation and imaging modalities. Traditionally, those steps are invariably fit for normal adults, but not pregnancy which requires certain limitations. We report this case of ileocolic intussusception secondary to an inflammatory fibroid polyp in a pregnant woman and describe our experiences in managing her.

## Case report

A 40-year-old pregnant mother, gravida 9 para 8 at 34-week gestation with underlying Beta-thalassemia carrier and hypertension presented to the Emergency Department with intermittent epigastric pain for 2 days. Pain onset was followed by recurrent loose stools and vomiting. She had had recurrent epigastric pain since 2013 however was never properly investigated. She was then admitted with an impression of acute dyspepsia in pregnancy. Upon presentation, she appeared lethargic, dehydrated, and tachycardic with a heart rate of 109 beats per minute. Her blood pressure was still within the normal range. Abdominal examination revealed a gravid uterus with tenderness over the epigastric region with no peritonism. Her biochemical investigations showed hypoalbuminemia with an albumin level of 18 (normal: 38–50 g/L), however, the other laboratory studies were within normal limits. An initial ultrasonography scan was performed which did not show any significant findings apart from nonspecific dilatation of the small bowel with mild ascites.

**Fig. 1:**
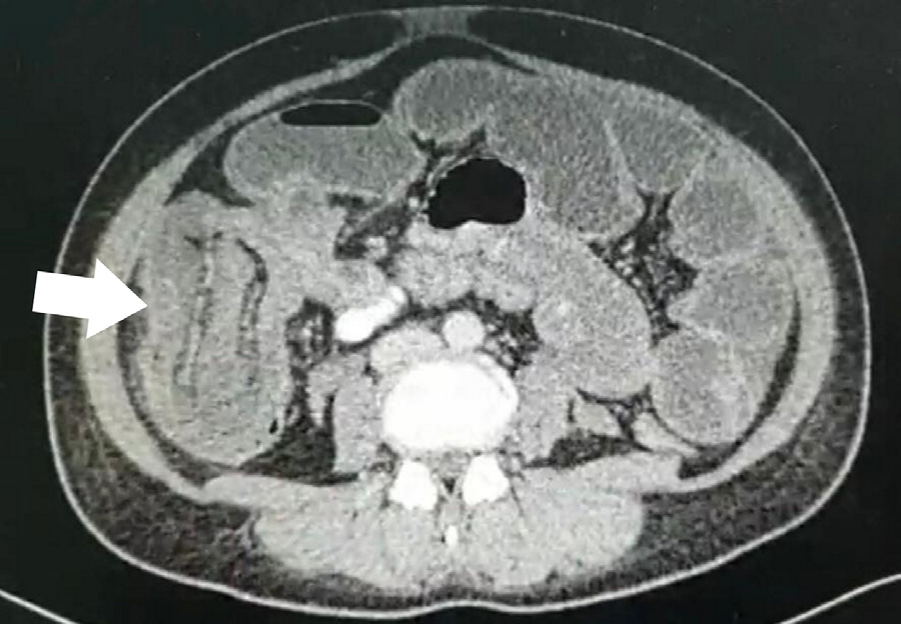
CT scan showing classical feature of elongated, sausage-shaped mass (arrow) in longitudinal view which represents an intussusception.

A day later, she passed out with minimal per rectal bleed associated with epigastric pain which spontaneously resolved. She denied any history of trauma or potentially contaminated food or water ingestion. She did not have any recent travel history or sick contact. She claimed the fetal kick chart was good and no per vaginal bleeding. Two days after the admission, she spontaneously delivered a healthy premature baby girl. Postdelivery, she was still complaining of recurrent epigastric pain which had migrated to the right flank. The patient eventually underwent abdominal computed tomography (CT) scan after the baby was delivered, which showed a long segment of small bowel intussusception (Fig. 1) up to the ascending colon.

**Fig. 2:**
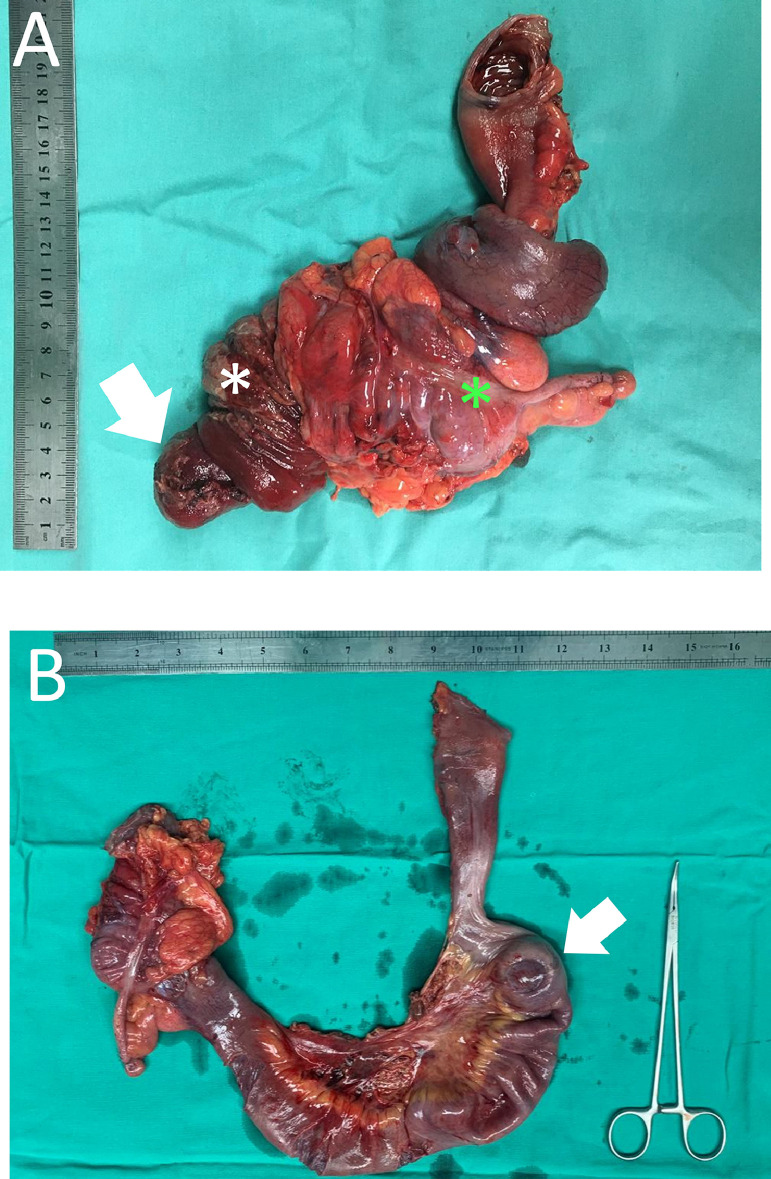
A right hemicolectomy (A) specimen showing a 3 × 3 cm rounded mass which represents the lead point (arrow). The mass and terminal ileum act as the intussusceptum (white *) telescope distally into the caecum (green *) that functions as the intussuscepient. The specimen (B) after being reduced revealed the full length of the invaginated small bowel measuring 40 cm long. The lead point (arrow) is markedly visualized as a rounded mass with indentation of the serosa.

**Fig. 3:**
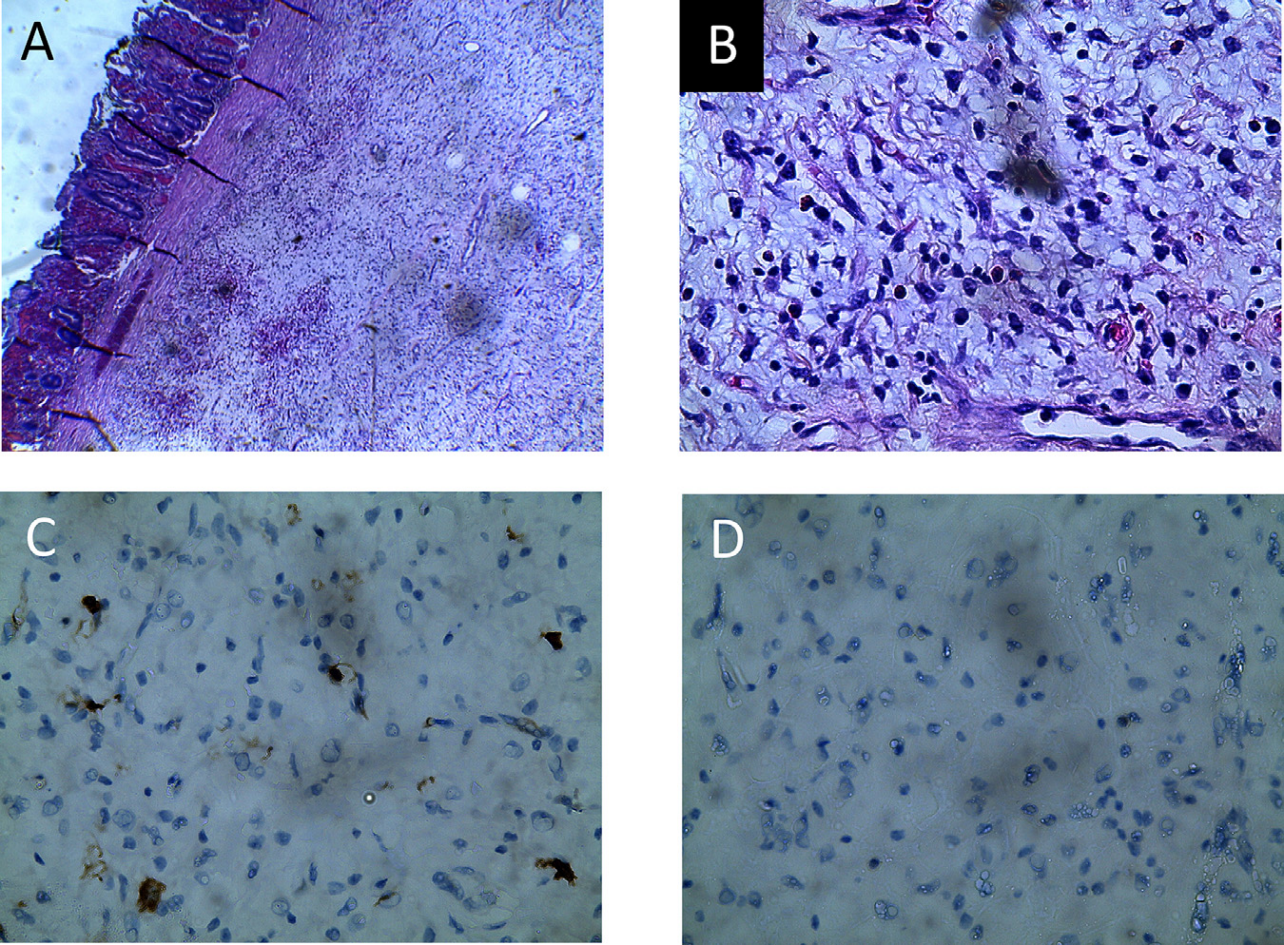
(A) Section shows a well-circumscribed submucosal lesion (Hematoxylin and eosin x4 magnification). (B) The lesion is composed of fairly uniformed spindle-to-stellate shaped cells with mucin in the background and mixed inflammatory cells infiltrates (Hematoxylin and eosin x40 magnification). (C) The cells show scattered positivity for CD117 immunohistochemistry (x40 magnification). (D) The cells are negative for ALK immunohistochemistry (x40 magnification).

We performed an emergency laparotomy on her after the CT scan, which revealed a segment of the ileum intussuscepted into the caecum and ascending colon. The trial of reduction was a failure and a segment of the small bowel in that area was seen as ischemic, hence we decided on right hemicolon resection with primary anastomosis. The postresection specimen showed a 5×5 cm small bowel tumor arising from the ileal mucosa, situated 30 cm proximally from the ileocecal valve (Fig. 2). Histopathology examination of the tumor was reviewed to be inflammatory fibroid polyp (Fig. 3) with ileocolic intussusception. Fortunately, her postoperative recovery was uneventful, she was discharged on the sixth postoperative day without any complications.

## Discussion

Intussusception in adults is rare, representing up to 0.03% of all hospital admissions, but, intussusception in pregnancy is even rarer [4]. Intestinal obstruction in pregnancy has been reported to occur with an incidence between 1:2500 and 3500, most commonly secondary to adhesions or gastrointestinal volvulus [5]. While in this case, it was due to an inflammatory fibroid polyp which is an extremely rare entity that arises within the submucosa of the gastrointestinal tract and represents less than 0.1% of all gastric polyps [6]. They are most commonly localized to the gastric antrum, small intestines and recto-sigmoid colon. Intestinal obstruction in pregnancy is associated with a maternal and perinatal mortality of 6% and 26%, respectively [7].

It is very challenging to diagnose intussusception during pregnancy. The presenting symptoms of nausea, vomiting, abdominal pain and constipation are common in pregnancy. In addition, the displacement of the bowel by the gravid uterus hampers the examination. Acute abdomen in pregnancy can be due to various causes including obstetric and non-obstetric causes. Thus, the diagnosis might be delayed. The majority of cases can present with obstructive symptoms and some are subtle symptoms, requiring meticulous clinical judgment with the use of radiological adjuncts to improve the diagnostic ability.

Various imaging modalities have been utilized to establish the diagnosis of acute abdomen. Among the most convenient modalities used include plain abdominal and chest radiography. The abdominal radiographs in the supine position can reveal distended bowel loops with haustration or/and valvulae conniventes which are typical for bowel obstruction. Meanwhile, the presence of classical air under the diaphragm in the erect chest radiography indicates a visceral perforation. However, both are generally unable to discern the etiology of the blockage secondary to intussusception. Ultrasonography, being a reliable and convenient diagnostic tool with radiation-free benefits, proves to offer superior advantages over radiography [8]. Features of the target sign in a transverse plane and pseudo-kidney sign in the longitudinal plane are diagnostic for intussusception. However, it is limited to obesity, intestinal obstruction with extensive bowel gasses, and operator dependent [8]. Among children, ultrasonography offers the best diagnostic modality with an adjunct for hydrostatic reduction, but it is different in adults [9].

CT scan is generally proven to be the most accurate investigation for intussusception with more than 80% accuracy [10,11]. It is commonly used in undifferentiated acute abdominal pain in general. A CT scan helps the radiologist with significant amounts of information. A bowel-within-the-bowel feature forming the target sign and sausage-shaped soft tissue mass is a classical finding of intussusception in CT scans. The lead point, if visible, can usually be identified as well. In addition, it provides the location, extension, and disease severity including the presence of any ischemia or perforation.

Another modality that can be chosen is magnetic resonance imaging (MRI). By using MRI on a fluid-sensitive sequence, an intussusception would demonstrate hyperintense intraluminal fluid with the low-to-intermediate signal intensity of the bowel wall with surrounding perienteric edema [12]. The lead points also can readily be visualized in MRI. However diagnostic imaging in pregnancy is restricted to ultrasound and MRI, with MRI not readily available in many hospitals. Furthermore, ultrasound was normal for this patient and this causes further delay in treatment. CT abdomen was performed postpartum and reviewed the evidence of intussusception.

The decision was made for emergency laparotomy for this lady and failed trial of reduction with evidence of bowel ischemia, bowel resection was then performed in this case. Adult intussusception necessitates surgical intervention because of the possibility of malignancy [13]. Controversy arises about whether to perform a reduction of an intussusception before resection to reduce the length of bowel resection to prevent short bowel syndrome. However, reduction of the intussuscepted bowel during surgery may result in seeding of the tumor in the abdominal cavity, perforation, abdominal infection, and anastomotic leakage [14].

## Conclusion

Acute abdomen in pregnancy can be due to various causes, thus a high index of suspicion and early CT abdomen might help in early diagnosis and treatment. The benefit of doing CT on the mother and the risk of CT on the fetus is to be weighed as the timely diagnosis can prevent bowel ischemia and reduce maternal morbidity and mortality. Surgery remains the definite management in adult intussusception and an exact diagnosis can be made during the operation. Reduction is not recommended as there is a high risk of malignancy in adult intussusception and bowel resection is recommended.

## Data availability statement

The data that support the findings of this study are available on request from the corresponding author. The data are not publicly available due to privacy or ethical restrictions.

## Patient consent

A written and informed consent for publication of this case was obtained from the patient.
